# A rare clinical presentation of plantar keratoderma

**DOI:** 10.11604/pamj.2025.52.4.48352

**Published:** 2025-09-01

**Authors:** Pawan Banduji Itankar, Gaurav Rajendra Sawarkar

**Affiliations:** 1 Department of Rachana Sharir, Mahatma Gandhi Ayurved College, Hospital, and Research Centre, Datta Meghe Institute of Higher Education and Research (Deemed to be University), Salod (H), Wardha, Maharashtra, India

**Keywords:** Keratoderma, callus, hyperkeratosis

## Image in medicine

A 56-year-old male agricultural worker from a tropical region presented with progressive thickening and painful callus-like lesions on the right sole over the past six months. He reported no history of trauma or systemic illness. Initially, he used over-the-counter moisturisers and emollients, which offered minimal relief. Over time, the lesions worsened, causing painful fissures and difficulty walking. A course of topical antifungals and steroids provided no lasting improvement. Clinical examination revealed diffuse hyperkeratosis with yellowish plaques and deep fissures on the soles. Based on history and findings, a diagnosis of plantar keratoderma was made. Plantar keratoderma is a disorder characterised by abnormal thickening of the plantar skin due to hyperkeratosis. It may be hereditary or acquired, commonly affecting individuals exposed to chronic mechanical stress, especially those walking barefoot or working in agriculture in tropical climates. Typical presentation includes painless or painful thickened soles, fissures, and restricted mobility. If untreated, the condition can lead to secondary infections and chronic discomfort. Management involves regular mechanical debridement, keratolytic agents such as salicylic acid or urea creams, and, in severe cases, systemic retinoids. Identifying any underlying cause or systemic association is important for long-term control. Unlike minor calluses, plantar keratoderma requires consistent dermatological care to prevent complications and improve quality of life.

**Figure 1:**
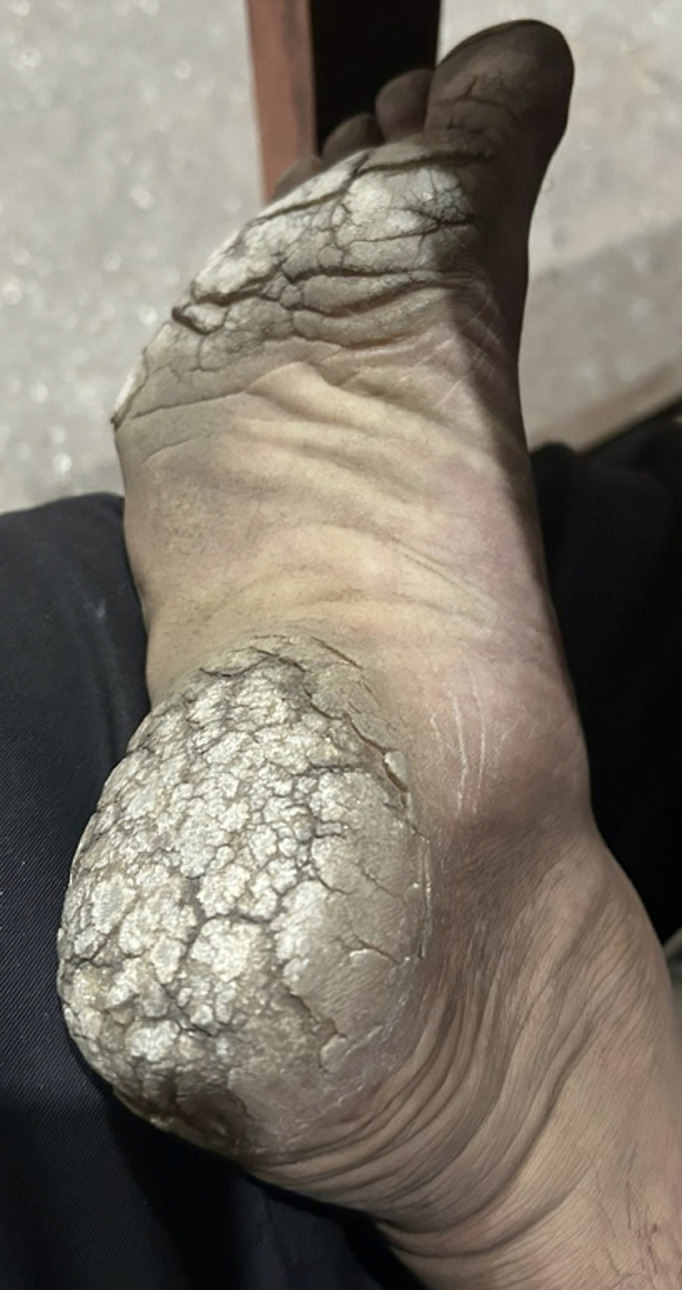
plantar keratoderma on the right sole

